# Supplementary material for: Human brain solute transport quantified by glymphatic MRI-informed biophysics during sleep and sleep deprivation
Source: Fluids Barriers CNS. 2023 Aug 18;20:62. doi: 10.1186/s12987-023-00459-8 (PMC10439559; doi:10.1186/s12987-023-00459-8)
Supplement: Supplementary file 1 — Additional file 1. Supporting Information for Human brain solute transport quantified by glymphatic MRI-informed biophysics during sleep and sleep deprivation. [file 12987_2023_459_MOESM1_ESM.pdf]

Additional file for Human brain solute transport quantified by glymphatic MRI-informed biophysics during sleep and sleep deprivation

Vegard Vinje[a], Bastian Zapf[b], Geir Ringstad[c,d], Per Kristian Eide[c,e], Marie E. Rognes[a], and Kent-Andre Mardal[a,b,1]

[a]Simula Research Laboratory, Kristian Augusts gate 23, 0164 Oslo, Norway
[b]Department of Mathematics, University of Oslo, Oslo, Norway
[c]Department of Neurosurgery, Oslo University Hospital – Rikshospitalet
[d]Department of Geriatrics and Internal Medicine, Sørlandet Hospital Arendal, Norway
[e]Institute of Clinical Medicine, Faculty of Medicine, University of Oslo, Oslo, Norway

Contents

S1 Evaluation of multi-modal data integration choices 1
S1.1 Mapping signal intensities to concentrations . . . 2
S1.2 Influence of using diffusion tensor images on simulation results . . . 5
S2 Numerics and computation 6
S2.1 Computational mesh characteristics . . . 6
S2.2 Numerical verification and convergence of the forward problem . . . 6
S3 Additional information for the inverse velocity field identification 8
S3.1 Numerical verification of the inverse velocity identification . . . 8
S3.2 Subject-level statistics of estimated convective flow field averages . . . 9
S3.3 Influence of DTI and T1 map on identified velocity fields . . . 9
S4 Additional information for the inverse identification of dispersion and reaction parameters 10
S4.1 Implementation details for inverse identification of dispersion and reaction parameters . . . 10
S4.2 Influence of DTI and T1 map on parameter identification . . . 10
S4.3 Numerical verification . . . 11
S4.4 Subject-level optimal enhanced diffusion-local clearance values . . . 11
References 11

S1 Evaluation of multi-modal data integration choices

The original clinical study[1], which data the present study rely on, involved 24 subjects. All subjects had T1-weighted MR images taken at multiple time points after intrathecal administration. 15 out of the 24 subjects had T_1 maps and 18 subjects had associated diffusion tensor images (DTI) taken before injection of tracers. Since not all data were available for all subjects, we first investigate whether raw, filtered or averaged T_1 maps and DTI data would yield quantitatively different conclusions in Section S1.1. Second, we assess the effect of using subject-specific DTI versus averaged diffusion coefficients on forward simulations, in Section S1.2. Finally, transport parameters that were obtained from PDE-constrained optimization are assessed with both subject-specific and study-averaged T_1 and DTI to confirm that parameters do not differ substantially between methods (Tables S6 and S7).

S1.1 Mapping signal intensities to concentrations

The T_1 maps enable conversion of contrast-enhanced MR signal intensities S (arbitrary unit) to tracer concentrations c (mmol/L). Specifically, for each subject, time of scan $t > 0$, and voxel a_i, we stipulate that:

$$c_{\mathrm{mri}}(a_i,t) = \frac{1}{r_1}\left(\frac{1}{T_1(a_i,t)} - \frac{1}{T_1^0(a_i)}\right), \tag{S1}$$

where T_1^0 is the longitudinal relaxation time prior to tracer injection as given by the T_1 maps, r_1 is a constant depending on the contrast agent, and $T_1(a_i,t)$ are the longitudinal relaxation times after tracer injection[2]. In agreement with[1], we normalize the contrast-enhanced signal intensities and compute

$$T_1(a_i,t) = f^{-1}\left(\frac{S(a_i,t)}{S_0(a_i)} f(T_1^0)\right), \tag{S2}$$

where S_0 is the pre-contrast T_1 weighted MRI signal and f is a monotone, nonlinear function specific to the MRI sequence used[3]. Parameters for the T_1 map acquisition using the MOLLI5(3)3[4] sequence, and hence the function f, are as previously established[3].

For 15 out of the 24 subjects in the present study, T_1 map images are available. The T_1 values are $900\,\mathrm{ms} \lesssim T_1 \lesssim 1400\,\mathrm{ms}$ in the parenchyma. Since the T_1 maps have lower resolution ($176 \times 176 \times 71$ voxels compared to $256 \times 256 \times 256$ in T_1 weighted images), significantly differing voxel value range, and are not available for all the participants, we investigate the importance of subject-specific T_1 map images for accurate quantification of tracer concentration in the brain. We consider three choices of T_1 maps for calculating the subject-specific concentrations, 1) using raw T_1 map, 2) using filtered T_1 map, and 3) using a group averaged T_1 map image. In detail, for the second method, for every of these 15 subjects, we create median-filtered T_1 maps by assigning the median T_1 map value of every subregion Ω_i (around 180 regions) as defined in the FreeSurfer parcellation file (wmparc.mgz)[5] to that region,

$$T_1(a_i) = \mathrm{median}_{y\in\Omega_i} T_1(y) \tag{S3}$$

where Ω_i indicates the FreeSurfer subregion that includes the voxel a_i. Finally, for the third method, we compute the average of the 15 available subjects as

$$\overline{T}_{1,k} = \frac{1}{15}\sum_{j=1}^{15}\frac{1}{\Omega_k^j}\sum_{a_i\in\Omega_k^j} T_1(a_i) \tag{S4}$$

where the index $k \in \{g,w,b\}$ indicates three brain subregions Ω_k cerebral cortex, subcortical subcortical white matter and the brain stem. This yields

$$\overline{T}_1(a_i) = \begin{cases} 1002\,\mathrm{ms} & \text{for } a_i \in \text{ subcortical white matter} \\ 1274\,\mathrm{ms} & \text{for } a_i \in \text{ cerebral cortex} \\ 1127\,\mathrm{ms} & \text{for } a_i \in \text{ brain stem} \end{cases} \tag{S5}$$

Using these mean values, as well as the filtered values (S3) and raw T_1 maps, we then estimate the concentration in every voxel a_i using (S1) and (S2) with $\overline{T}_1$. The voxel-based concentration values $c_{\mathrm{mri}}(a_i,t)$ are then used to represent the brain-wide concentration field $c(x,t)$ on the computational mesh using continuous linear Lagrange elements. The total amount of tracer imaged in the brain at time t is computed as

$$\int_{\Omega} c(x,t)\,\mathrm{d}x \tag{S6}$$

and the total amount of tracer imaged on the brain surface at time t, is computed as

$$\int_{\partial\Omega} c(x,t)\,\mathrm{d}S. \tag{S7}$$

The resulting concentration values in the brain and on the surface are displayed in Figs. S1A-B. The three T_1 maps yield average brain concentration around 0.1 mmol/L after 6 and 24 hours, and there are no significant differences between the choices neither in concentration in the brain nor at the surface (at all time points). The estimates differ the most at 24 h; in the brain the concentration differs by $7 \pm 2\,\%$ between method 1 and 2, and $11 \pm 4\,\%$ by 2 and 3. At the pial surface, the values

differ by $13 \pm 3\,\%$ between method 1 and 2, and $24 \pm 7\,\%$ by 2 and 3. Fig. S1C compares simulation and data for the three choices of T_1 map. The conclusion drawn from either choice is the same; simulations underestimate the influx at $\sim 6\,\mathrm{h}$ by $59, 61, 58\,\%$ and overestimate what remains in the brain after $48\,\mathrm{h}$ by $81, 93, 85\,\%$ for the T_1 maps 1-3, respectively.

Across the subjects, the resulting concentrations correlate strongly with the MRI signal increase inside the brain (0.97-0.99) and at the pial surface (0.89-0.99), cf. Fig. S1D-K. Further, the concentrations obtained from averaged T_1 maps increase correlate strongly with those obtained by raw T_1 map, (0.98-1.00) for brain-concentrations and (0.97-0.99) at the pial surface, cf. Fig. S1L-S.

These observations justify the use of synthetic T_1 maps $\overline{T}_1$ to estimate the concentration in the 9 / 24 subjects where no T_1 map is available. We hence use method 3 (group averaged T_1 map image) to estimate the concentration in all 24 subjects.

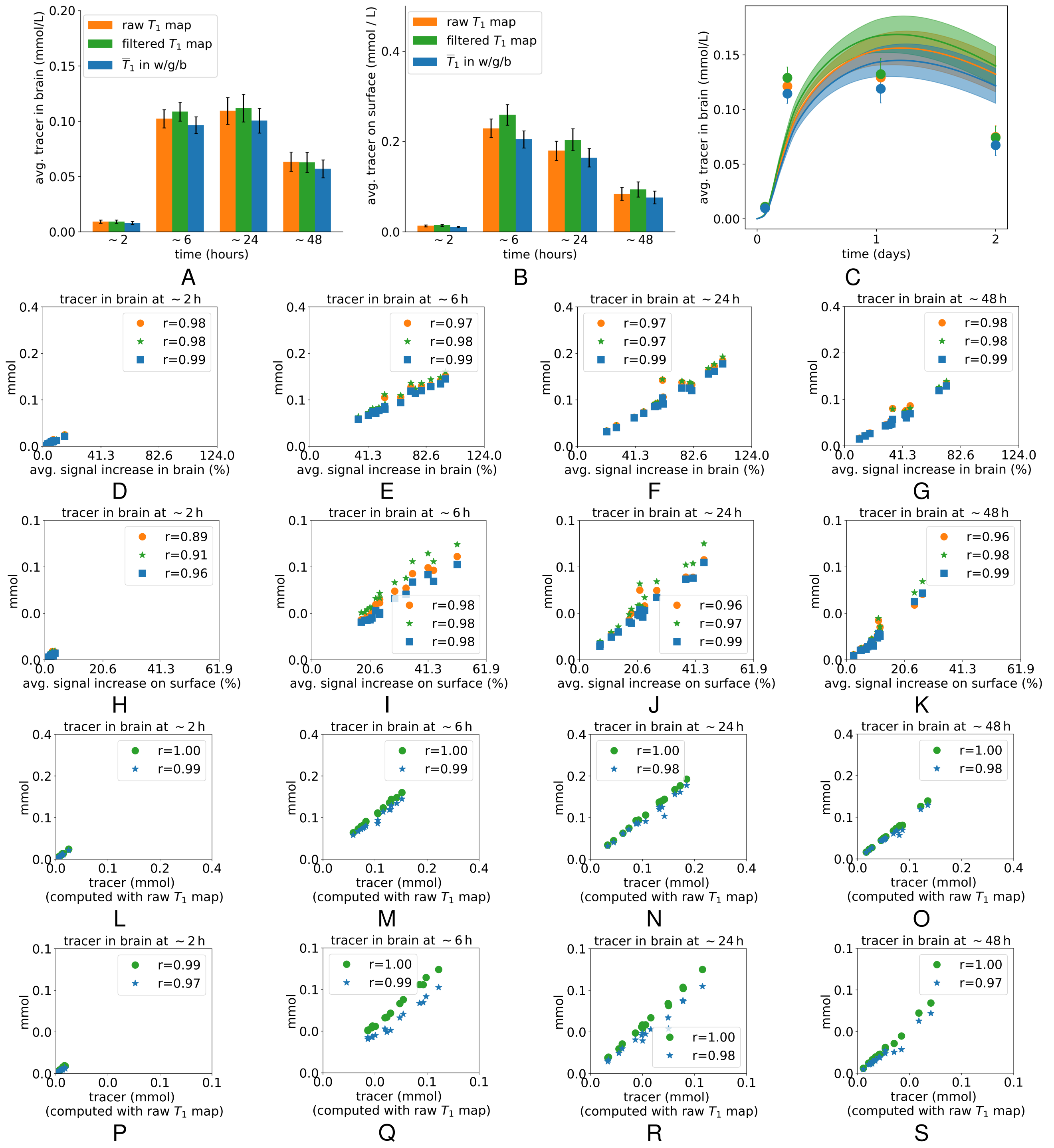

Figure S1. Tracer in brain (A) and at the pial surface (B) as computed with the three different choices for T_1 maps. (C) Measured (dots) and simulated (continuous lines) tracer in the brain, averaged over the $n = 15$ subjects where good T_1 maps are available. Error bars and shaded regions indicate standard error of the mean. (D-G) correlation between signal increase and tracer in the brain for different time points. (H-K) correlation between signal increase and tracer at the pial surface for different time points. (L-O) correlation between tracer as computed with raw T_1 map and computed with the other two methods, in the brain, for different time points. (P-S) correlation between tracer as computed with raw T_1 map and computed with the other two methods, at the pial surface, for different time points.

S1.2 Influence of using diffusion tensor images on simulation results

From the DTI scans, we directly obtain subject-specific voxel-wise apparent diffusion tensors for water $\mathbf{D_0^*}$. The apparent diffusion coefficient for water D_0^* is computed as the mean diffusivity:

$$D_0^* = \frac{1}{3}\mathrm{tr}(\mathbf{D_0^*}).$$

Given also the free diffusion coefficient of water D_0 we estimate the tortuosity of the brain as $\lambda^2 = D_0/D_0^*$, and (assuming the tortuosity for water and CSF tracer is the same) the apparent diffusion coefficient of the CSF tracer is thus given by:

$$D^* = \frac{D}{\lambda^2} = \frac{D}{D_0}D_0^*,$$

where $D = 3.8\times10^{-4}\,\mathrm{mm^2 s^{-1}}$ is the free diffusion coefficient of the CSF tracer gadobutrol[3] used in this study. To convert diffusion tensors from water to CSF tracer diffusivity, we correspondingly scale the DTI values by the ratio D/D_0 to obtain the diffusion tensor in each voxel a_i:

$$\mathbf{D}^*(a_i) = \frac{D}{D_0}\mathbf{D_0^*}(a_i). \tag{S8}$$

For 18 out of 24 subjects, DTI taken at baseline (before tracer injection) were available. We first checked if the mean diffusivities estimated from these images differs between the sleep and sleep deprivation group. From the diffusion tensor $\mathbf{D}_0^*$ for water we computed the averaged mean diffusivity $\int \frac{1}{3}\mathrm{tr}(\mathbf{D}_0^*)\,\mathrm{d}x$ in subcortical white matter and the cerebral cortex for every subject. The values are illustrated in Fig. S2. No significant differences between groups were found in either subcortical white matter or the cerebral cortex.

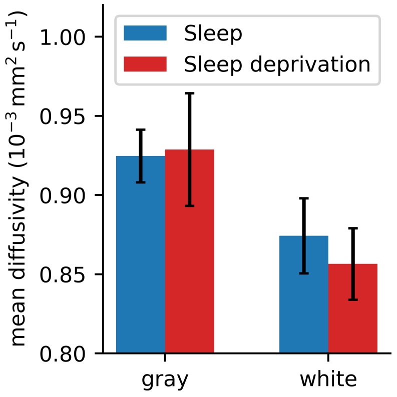

Figure S2. The average water diffusivity in subcortical white matter and the cerebral cortex does not vary significantly between the two subject groups. Error bars represent standard error of the group mean.

For 6 out of the 24 subjects, no DTI was available. In order to incorporate these subjects into the analysis, we investigated the feasibility of using mean scalar diffusion coefficients instead of subject-specific DTI for the diffusivity D^* in (1). From the $j = 1,\dots,18$ subjects where DTI was available, we computed study average mean scalar diffusion coefficients as

$$\overline{D}_i = \frac{1}{18}\sum_{j=1}^{18}\frac{1}{\Omega_i^j}\int_{\Omega_i^j}\frac{1}{3}\mathrm{tr}(\mathbf{D}_0^*)\,\mathrm{d}x \tag{S9}$$

where the index $i \in \{g,w,b\}$ indicates three brain subregions Ω_i^j cerebral cortex, subcortical white matter matter and the brain stem of subject j. Using these values, we then solve (1) with D as defined in (S9),

$$D(x) = \begin{cases} \overline{D}_g = 1.17\times10^{-4}\,\mathrm{mm^2 s^{-1}} & \text{for } x \in \text{ cerebral cortex} \\ \overline{D}_w = 1.10\times10^{-4}\,\mathrm{mm^2 s^{-1}} & \text{for } x \in \text{ subcortical white matter matter} \\ \overline{D}_b = 1.34\times10^{-4}\,\mathrm{mm^2 s^{-1}} & \text{for } x \in \text{ brain stem} \end{cases}$$

as well as the spatially varying diffusion tensor (S8). The resulting simulated concentration distributions after 48 h are shown for an example subject in Fig. S3. Both choices lead to visually identical predictions, and in the shown example, the relative l^1-difference is 7 %.

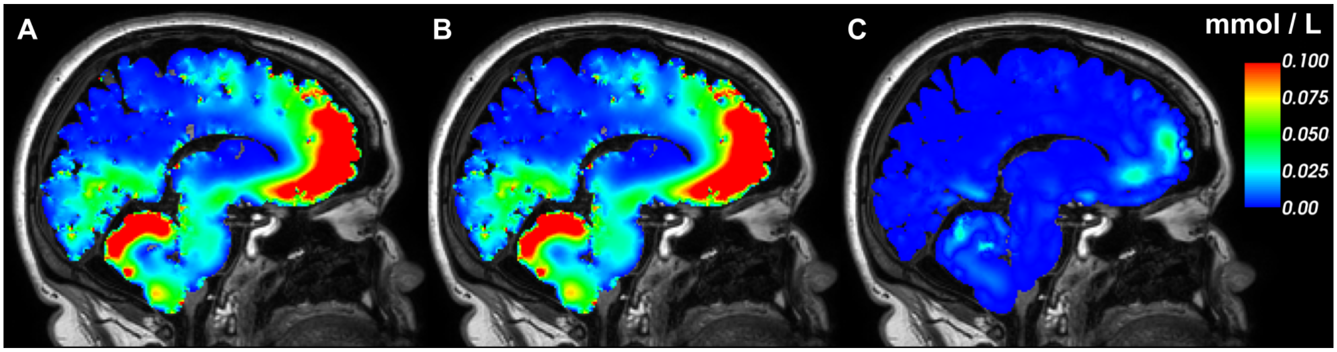

Figure S3. Simulated tracer distribution after 48 hours in example subject 14, using DTI (A) and averaged diffusion coefficients (B). The absolute error between A-B is shown in (C) in the same color scale.

Fig. S4A illustrates the tracer concentration over time as simulated with both choices averaged over the 18 subjects with DTI. The relative difference between the curves is about 1 %. In both cases, the amount of simulated tracer after 5-7 h is about 60 % too small as compared to data. After two days it is 200 % too large in both choices.

Figs. S4B-D further show the correlation between tracer in the brain as measured and simulated using DTI/simulated with averaged D at 6, 24, 48 hours. Overall, the correlation is in the range (0.91-0.95). At every time point, the correlation measures for both methods are nearly identical.

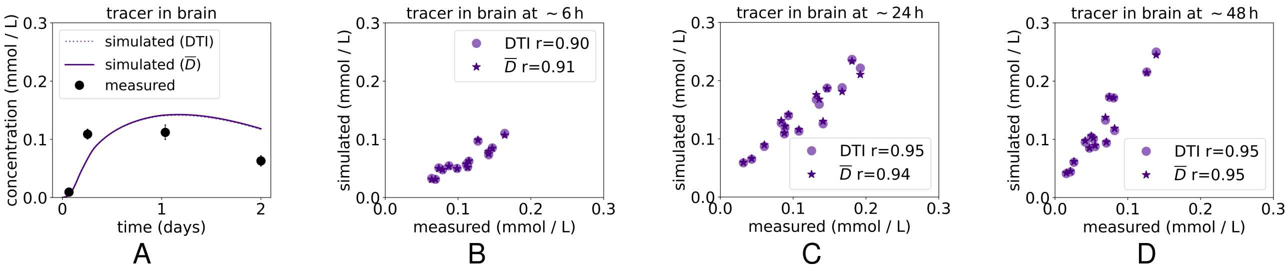

Figure S4. A. Tracer in brain as measured (dots) and simulated with DTI and avg. $\overline{D}$ (continuous lines) over two days. B-D. Correlation between tracer in the brain as measured (horizontal axis) and simulated using DTI / simulated with averaged D (vertical axis) at 6, 24, 48 hours for the subjects where DTI is available.

In summary, the difference in simulated averaged concentrations between choosing an average diffusion constant versus using the diffusion tensor from DTI differed by less than 1 % (Fig. S4a). Furthermore, the correlation coefficients between the two methods were all $r = 1.00$ at 6, 24 and 48 hours. Based on these results, in each of the 24 subject we choose to assign the mean diffusion coefficients given by (S10) to a given region (cerebral cortex, subcortical white matter matter and brain stem).

S2 Numerics and computation

S2.1 Computational mesh characteristics

For each subject, left and right hemisphere pial surfaces, left and right cerebral cortex/subcortical white matter interface surfaces as well as ventricular surfaces were extracted from the T1-weighted MR images prior to tracer injection using FreeSurfer[6]. The surfaces were refined and smoothened using computational geometry techniques prior to construction of volumetric meshes of the brain parenchyma at low-, standard- and high resolution via SVMTK[7]. Table S1 gives an overview of the complexity and approximability of the meshes at different resolution levels.

S2.2 Numerical verification and convergence of the forward problem

In order to access the numerical convergence of the forward diffusion simulations, we performed simulations of (1) using different mesh resolutions and time step sizes. In detail, for two example subjects, we solve (1) over a time span of two days with $\alpha = 1, r = 0, \phi = 0$ with time steps of 30, 20, 10 min and meshes of resolution parameter 64, 32, 16 (corresponding to maximal mesh cell sizes of $\sim 3, 5, 11$ mm, respectively).

subject ID	Low-res				Standard				High-res			
	n_v	n_c	$h_{\min}$	$h_{\max}$	n_v	n_c	$h_{\min}$	$h_{\max}$	n_v	n_c	$h_{\min}$	$h_{\max}$
1	56663	268451	0.21	10.42	205649	917870	0.10	5.23				
2	75080	374297	0.40	10.70	213735	1012961	0.31	5.41				
3	73549	385438	1.05	10.88	247863	1167963	0.52	5.47	9456	49843	2.42	23.81
4	76920	392357	1.01	10.99	229201	1085103	0.52	5.51	864112	4044782	0.25	2.77
5	78000	394201	1.10	11.02	229561	1085115	0.43	5.55				
6	74747	404183	1.06	10.66	250598	1205522	0.45	5.37				
7	80552	421045	0.94	11.01	250010	1192453	0.09	5.43	971769	4545533	0.13	2.73
8	73893	376981	0.96	10.30	236044	1103313	0.45	5.18	925360	4327308	0.24	2.60
9	74305	380830	0.89	10.26	230342	1086651	0.50	5.14	899409	4205827	0.15	2.58
10	79983	403220	0.98	10.59	228810	1080081	0.43	5.38	855631	4023766	0.23	2.69
11	80824	404862	0.60	10.81	228540	1075838	0.52	5.42	869291	4044744	0.10	2.73
12	86192	448242	0.87	10.95	256069	1225482	0.48	5.49	973581	4554988	0.24	2.75
13	81574	424664	1.00	10.83	253784	1199128	0.51	5.43	955595	4428214	0.04	2.72
14	81143	423197	1.02	10.91	240837	1167721	0.50	5.47	950203	4453739	0.24	2.75
15	79428	406316	1.01	10.90	234481	1117785	0.54	5.58	903415	4243049	0.23	2.77
16	79059	405030	0.94	10.69	239084	1136336	0.51	5.40	915324	4314036	0.02	2.70
17	80163	415968	1.04	11.06	239221	1141950	0.55	5.60	925175	4293556	0.03	2.81
18	78527	391206	0.95	11.37	211243	1006364	0.47	5.73	766404	3613485	0.24	2.87
19	78125	400132	1.04	10.67	238326	1129354	0.47	5.35	926311	4339819	0.16	2.68
20	76058	379266	1.10	10.46	220072	1038529	0.50	5.31	829541	3944186	0.23	2.66
21	82373	420395	1.00	10.40	251776	1188577	0.47	5.22	954926	4500535	0.22	2.61
22	82737	425841	0.96	10.79	250220	1184843	0.48	5.43	1039209	4761980	0.23	2.72
23	81636	404870	0.97	10.62	234802	1104395	0.47	5.35	882364	4163055	0.22	2.70
24	75769	390828	0.95	10.37	240209	1140383	0.46	5.19				

Table S1. Overview of computational mesh characteristics for each subject for low- standard and high-resolution meshes. Here, n_v denotes the number of mesh vertices, n_c the number of mesh cells, and the minimal and maximal mesh cell sizes $h_{\min}$ and $h_{\max}$ (in mm) are computed as the minimal and maximal mesh cell circumradius $\times 2$, respectively.

Fig. S5 shows the simulated brain-wide CSF tracer concentration over a time span of two days for the two example subjects. The low resolution meshes yield visibly lower concentrations compared to standard and high resolution meshes; integrated concentrations differ to a relative error of 14 % and 20 % between low and high resolution meshes for example subject 4 and 14, respectively. However, standard and high resolution meshes yield comparable results, the simulated concentrations differ to a relative error of 1 % and 4 % for example subject 4 and 14, respectively.

The errors between simulated tracer with low and high resolution meshes and time step of 20 min are 15 % after 48 hours for subject 4, and 19 % after 48 hours for subject 14. For standard and high resolution meshes, the errors between simulated tracer after 48 hours are 1 % after 48 hours for subject 4, and 3 % for subject 14, indicating that the conclusion drawn from either standard or high resolution mesh is the same.

The data shown in Table S2 show that the simulated concentration after 48 h agrees up to the third digit for standard and high resolution meshes and all time step sizes. We hence conclude that standard resolution meshes yield accurate results and use meshes of this resolution to produce the results reported in the main manuscript. For the inverse velocity identification we perform simulations with low resolution meshes in addition to investigate the convergence of the method, cf. Section S3.1. For the forward diffusion simulations with $\alpha \in \{1,2,3,4,5\}$ and $r = \phi = 0$, we use a time step size of 20 min to limit the computational cost.For the inverse identification of parameters in the enhanced diffusion–local clearance model, we assess the influence of time step size on the results separately in Section S4.3.

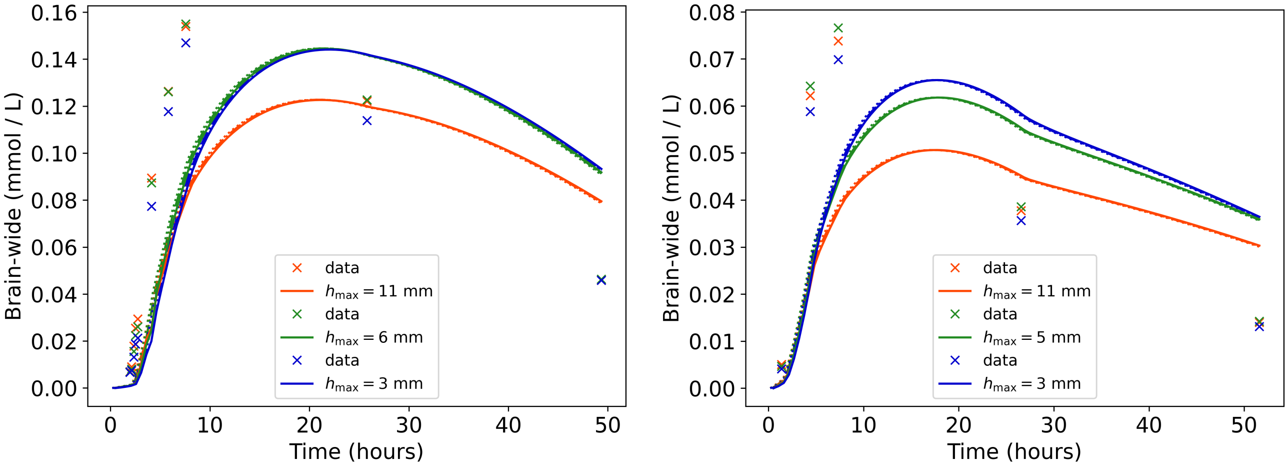

(a) Example subject 4 **(b)** Example subject 14

Figure S5. Dependence of the tracer concentration obtained by solving (1) with $\alpha = 1, r = 0, \phi = 0$ using meshes of different resolution. The different time step sizes of 30, 20 and 10 min yield similar results and are encoded by line styles "-", ".-" and "..", respectively.

S3 Additional information for the inverse velocity field identification

This section gives additional methodological details and results for the inverse estimation of the velocity field ϕ in the following special case of (1) (with $\alpha = 1, r = 0$):

$$\frac{\partial c}{\partial t} - \nabla \cdot D^* \nabla c + \nabla \cdot (c\phi) = 0 \quad \text{in } \Omega, \quad t > t_0. \tag{S10}$$

S3.1 Numerical verification of the inverse velocity identification

In order to evaluate the numerical accuracy of the computationally estimated velocity fields, we considered a series of experiments for different computational mesh resolutions (low and standard resolutions) and different regularization parameters ($\beta \in \{10^{-3}, 10^{-4}, 10^{-5}\}$) for each subject and for each of the time intervals 0–6h, 6–24h and 24–48h. For each of these simulations, we first evaluated the robustness and convergence of the iterative optimization algorithm by comparing the value of the initial functional J_0 and optimized functional J_N $N = 80$), the maximal component of the final (optimized) velocity field $\max \phi$, and the relative difference between the two last values of the maximal velocity (see Table S3 for the time interval 24–48h). Two subjects (3, 23) were missing contrast MR scans in the ∼6h time interval, while three subjects (1, 2, 5) where missing scans in the 24–48h interval. These subjects were therefore excluded from further analysis in the relevant time interval.

For the $\sim$0–6h intervals, the objective functionals were substantially reduced for all remaining subjects and numerical parameters (n, β), typically by two–three orders of magnitude (by a factor ranging from 6 to 614), and the relative differential in the maximal velocity component was less than 1.84% for all cases. Similarly, for the $\sim$6–24h intervals, all optimizations were considered satisfactory with objective functional reductions of $26-4765\times$ and a maximal relative differential of 1.06% (data not shown). For the $\sim$24-48h intervals, the optimization algorithm did not converge satisfactorily for subject IDs 6, 12 and 14 for any β, n (little to no reduction in the objective functionals). For these artefactual cases, the maximal velocity component estimates also varied substantially with β. We therefore excluded the numerical results also from these subjects for the interval 24–48h from the subsequent analyses. For the remaining subjects, the maximal relative differential in the maximal velocity component was 2.14% and the relative reduction in the objective functional substantial (8–2098$\times$).

We next proceeded to evaluate the numerical accuracy of the optimal velocity estimates, in particular, we are interested in any dependence of the numerical results on the primary numerical parameters such as the mesh resolution n and choice of regularization parameter β. For each numerical simulation (having passed the optimization algorithm evaluation described above), we computed the average velocity magnitude $\bar{\phi}$ for each velocity field $\phi = \phi(x) = (\phi_1(x), \phi_2(x), \phi_3(x))$ as

$$\bar{\phi} = |\Omega|^{-1} \int_{\Omega} |\phi| \,\mathrm{d}x, \quad |\Omega| = \int_{\Omega} 1 \,\mathrm{d}x, \quad |\phi|^2 = \phi_1^2 + \phi_2^2 + \phi_3^2,$$

as well as the L^2-norm of the associated estimated concentration field $c = c(x,t_1)$ solving (1) on the relevant time interval (t_0,t_1), the L^2-norm of the corresponding target concentration c_1, and the relative difference between these values Δt. Based on the following analysis of these values, we use $n = 32$ and $\beta = 10^{-4}$ for the numerical simulations reported in the main text.

For the first time interval, $\sim$0–6h (data not shown), we observe that the relative difference in concentration norms is small for all experiments (typically less than 1%, with a maximal relative difference when $\beta = 10^{-4}$ of 3.84%). Moreover, we observe that the estimated average velocity shows only small variations (if any) with respect to the regularization parameter β especially for the standard resolution meshes $n = 32$. However, we do observe some variations with respect to the mesh size n, indicating that a comparison with a high resolution mesh could give further insights with regard to the numerical convergence of the method. For the time interval $\sim$6–24h (data not shown), we also observe low relative differences in concentration norms for all experiments: maximal relative difference when $\beta = 10^{-4}$ is 0.83%. The average velocity is highly robust with respect to β, and also its variation with respect to n is relatively small.

For the time interval $\sim$24–48h (Table S4), we observe higher relative differences in concentration norms for $\beta = 10^{-3}$ (up to 10%), but the relative difference remains low for $\beta = 10^{-4}, 10^{-5}$. The maximal relative difference for $\beta = 10^{-4}$ is 1.63%. We observe some variation in the average velocity norm for $\beta = 10^{-3}$, while variation is less when comparing $\beta = 10^{-4}$ and 10^{-5}. We observe some, but generally only small, variations between mesh sizes, and thus consider $\beta = 10^{-4}$ and $n = 32$ to yield more accurate numerical solutions.

S3.2 Subject-level statistics of estimated convective flow field averages

The subject-specific averaged velocity magnitudes, averaged brain-wide (v), over the cerebral cortex (v_g), over the subcortical white matter matter (v_w) and over the brain stem (v_s) are given in Table S5 for 6–24h (top) and 24–48h (bottom). The average velocities in the intervals 6–24 h and 24–48h correlate only weakly with $r \approx 0.3$ in all regions of interest, cf. Fig. S6.

As shown in Table S5, we find that for the reference group at 6–24 hours, the regional average flow speeds mutually differ (paired t-test, p-values for cerebral cortex-subcortical white matter: 0.00016, cerebral cortex-brain stem: 0.011, white-brain stem: 0.0018). For the sleep-deprived group at 6–24h, the regional average flow speeds mutually differ, except when comparing the cerebral cortex/brain stem (paired t-test, p-values for cerebral cortex-subcortical white matter: 0.0018, cerebral cortex-brain stem: 0.075, subcortical white matter-brain stem: 0.031). The brain-wide and regional flow speeds do not differ between groups at 6h (Welch's t-test, p-values: 0.34, 0.40, 0.24, 0.79). For the groups combined, at 6–24h, the regional average velocities mutually differ (paired t-test, p-values for cerebral cortex-subcortical white matter: 1.1e-06, cerebral cortex-brain stem: 0.0013, subcortical white matter-brain stem: 8.9e-05). For the reference group at 24–48h, the subcortical white matter and cerebral cortex average flow speeds differ (paired t-test, p-values for cerebral cortex–subcortical white matter: 0.0031, cerebral cortex–brain stem: 0.23, subcortical white matter–brain stem: 0.87). For the sleep-deprived group at 24–48h, the subcortical white matter and cerebral cortex average flow speeds differ (paired t-test, p-values for cerebral cortex–subcortical white matter: 0.037, cerebral cortex–brain stem: 0.46, subcortical white matter–brain stem: 0.93).

S3.3 Influence of DTI and T1 map on identified velocity fields

We further assessed how sensitive the identified velocity fields are with respect to data integration choices. Using (a) subject-patient specific diffusion tensors and T_1 maps and (b) study averaged constants for D and T_1 for the 15 subjects where this data is available, we compare quantities of interest of the resulting velocity field obtained with regularization parameter $\beta = 10^{-4}$. The quantities differ to no more than 3% between the data integration choices (a) and (b), with the brain stem being an exception,

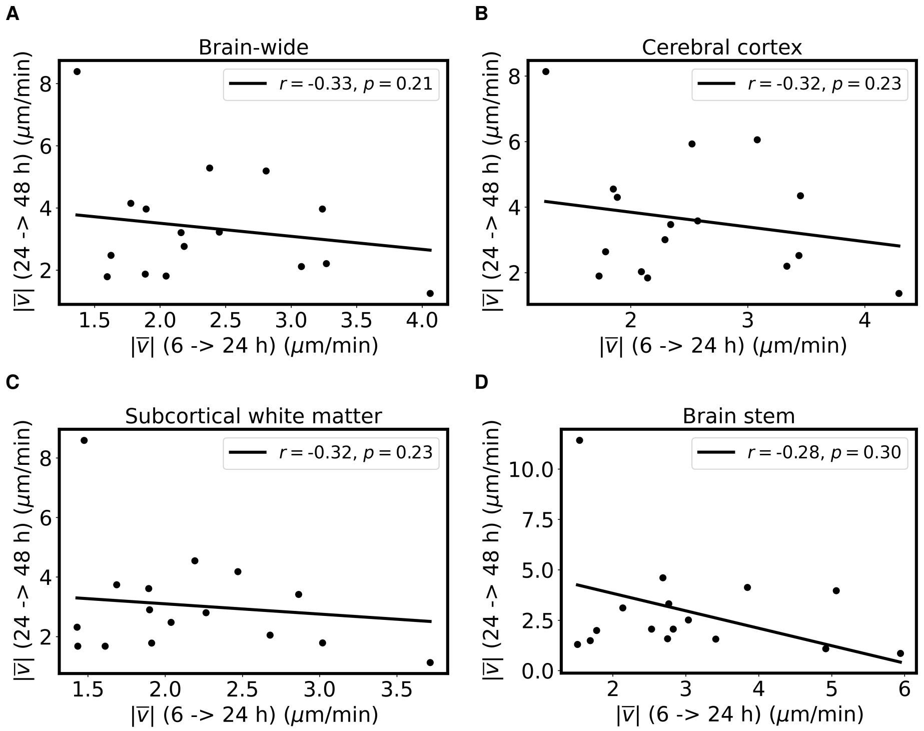

Figure S6. Correlation between average velocities in 6-24 h interval to 24-48 h interval.

where the maximum difference between (a) and (b) is the fluid inflow rate from 6 to 24 h and it differs to 24 %. Overall, the results presented in Table S6 suggest that the quantities of interest computed from the identified velocity fields are robust with respect to data integration choices. This justifies using study averaged constants for D and T_1 for the 9 subjects were this data is not available.

S4 Additional information for the inverse identification of dispersion and reaction parameters

This section gives additional methodological details and results for the inverse estimation of dispersion-reaction parameters, i.e. we consider finding α and r in the following special case of (1) with $\phi = 0$

$$\frac{\partial c}{\partial t} - \nabla \cdot \alpha D^* \nabla c + rc = 0 \quad \text{in } \Omega, \quad t > t_0 \tag{S11}$$

such that c solves the PDE and minimizes the mismatch to the MRI observations.

S4.1 Implementation details for inverse identification of dispersion and reaction parameters

In order to solve (1) numerically, the equation is discretized in time via the Crank-Nicolson method and in space via the finite element method with continuous linear Lagrange elements. We find that for most subjects, a time step of 10 min yields convergence in the estimated dispersion and clearance parameters (cf. Section S4.3), but in some subjects (cf. Table S9), we performed additional simulations with time step size 5 min to ensure that the algorithm had converged. To obtain good starting values for the optimization of α, r we perform a grid search with combinations $\alpha \in \{1,2,3,4,5\}$, $r \in \{0, 6.25 \times 10^{-6}, 1.25 \times 10^{-5}, 2.5 \times 10^{-5}, 3.54 \times 10^{-5}, 5 \times 10^{-5}, 7.07 \times 10^{-5}, 10^{-4}\}$ for every subject. We find $\alpha = 3, r = 10^{-5}\,\text{s}^{-1}$ as parameters that generally lead to significantly better agreement between simulations and data, and hence start the PDE-constrained optimization with these values for all subjects.

S4.2 Influence of DTI and T1 map on parameter identification

We further assessed how sensitive the identified dispersion-reaction parameters (α, r) are with respect to data integration choices. Using (a) subject-patient specific diffusion tensors and T_1 maps and (b) study averaged constants for D and T_1 for the 15 subjects were this data is available, we compare optimal parameters obtained from PDE constrained optimization with a

time step of 10 min in Table S7. For α, the variation between methods is 0.002 which is much smaller than the variation ~ 1.1 between subjects, as measured by the standard deviation. With respect to the local clearance parameter r, the methods differ to $3.4 \times 10^{-4}\text{min}^{-1}$ which is still one order of magnitude smaller than the variation between subjects ($\sim 15 \times 10^{-4}\text{min}^{-1}$). Overall, the similarity of subject-specific optimal parameters presented in Table S7 suggest that both α and r are robust with respect with respect to method (a) and (b). This justifies using study averaged constants for D and T_1 for the 9 subjects were this data is not available.

S4.3 Numerical verification

In order to ensure convergence of the PDE-constrained optimization algorithm with respect to the time step size used in the forward simulation, we performed computations with time step size of 30, 20 and 10 min for every subject. The results are given in Table S8. While the best parameters change between time step size 30 and 10 min, for most patients, the optimal parameters α and r differ to no more than 10% between time step sizes 20 and 10 min. We hence consider the algorithm to be converged in these subject, and report the results obtained with time step 10 min in the manuscript. In those subjects where this criterion was not met, we perform additional simulations with time step size 5 min to ensure that the algorithm has converged. We find that in these subjects, the resulting α and r differ to no more than 10% between time step sizes 10 and 5 min. We thus consider the algorithm to be converged in these subjects, and use the results obtained with time step size 5 min in these cases. For subject ID 3, the optimal parameters change substantially between a time step size of 20 and 10 minutes. This is due to the fact that considerably many images in the first hours after injection are available for this subject as compared to the other subjects in this study. In detail, images taken every 10 minutes in the time between two and three hours after injection are available for this subject. Since the boundary condition is taken as a linear interpolation between images, a time step size of 20 minutes hence is too large for this data set. For this subject, the optimization algorithm converges to the upper bound $\alpha = 10$, and we hence excluded this subject from the results reported in the main text.

It should be noted that optimization for one subject typically requires around 15, 23, 49, and 81 hours for time steps 30, 20, 10 and 5 min, respectively, on a supercomputer node with 12 CPU cores.

S4.4 Subject-level optimal enhanced diffusion-local clearance values

Table S9 summarizes the results from PDE-constrained optimization for the best enhanced diffusion-local clearance parameters α, r for all subjects using averaged diffusion coefficients and T_1 values. The best parameters reduce the relative L2 mismatch to the MRI observations about $24 \pm 9\,\%$ (from $32 \pm 5\,\%$ to $24 \pm 6\,\%$) compared to diffusion simulations.

References

References

1. Eide, P. K., Vinje, V., Pripp, A. H., Mardal, K.-A. & Ringstad, G. Sleep deprivation impairs molecular clearance from the human brain. *Brain* **144**, 863–874 (2021).
2. Rohrer, M., Bauer, H., Mintorovitch, J., Requardt, M. & Weinmann, H.-J. Comparison of magnetic properties of mri contrast media solutions at different magnetic field strengths. *Investig. radiology* **40**, 715–724 (2005).
3. Valnes, L. M. *et al.* Apparent diffusion coefficient estimates based on 24 hours tracer movement support glymphatic transport in human cerebral cortex. *Sci. reports* **10**, 1–12 (2020).
4. Taylor, A. J., Salerno, M., Dharmakumar, R. & Jerosch-Herold, M. T1 mapping: basic techniques and clinical applications. *JACC: Cardiovasc. Imaging* **9**, 67–81 (2016).
5. Destrieux, C., Fischl, B., Dale, A. & Halgren, E. Automatic parcellation of human cortical gyri and sulci using standard anatomical nomenclature. *Neuroimage* **53**, 1–15 (2010).
6. Fischl, B. Freesurfer. *Neuroimage* **62**, 774–781 (2012).
7. Mardal, K.-A., Rognes, M. E., Thompson, T. B. & Valnes, L. M. Mathematical modeling of the human brain: From magnetic resonance images to finite element simulation (2022).

Table S2. Brain-wide CSF tracer concentration (mmol / L) at 48 hours, data and diffusion simulation ($\alpha = 1, r = 0, \phi = 0$), for different time/mesh resolutions for two example subjects.

	dt	$n = 16$	$n = 32$	$n = 64$
data	-	0.046	0.046	0.046
simulation	30	0.101	0.118	0.119
	20	0.101	0.118	0.119
	10	0.100	0.117	0.118

(a) Example subject 4

	dt	$n = 16$	$n = 32$	$n = 64$
data	-	0.014	0.014	0.013
simulation	30	0.037	0.045	0.046
	20	0.037	0.045	0.046
	10	0.037	0.045	0.046

(b) Example subject 14

ID, t0	n	J_0	J_N	$\max\phi_N$ ($\Delta\phi$%)	J_0	J_N	$\max\phi_N$ ($\Delta\phi$ %)	J_0	J_N	$\max\phi_N$ ($\Delta\phi$ %)
3, 24h	16	1482.9	146.52	3.27 (0.44)	1482.9	65.39	3.79 (0.18)	1482.9	39.86	4.28 (0.10)
	32	1450.4	143.04	2.24 (0.53)	1450.4	73.71	6.17 (0.03)	1450.4	54.00	8.05 (0.40)
4, 24h	16	5050.8	106.96	2.17 (1.53)	5050.8	26.67	3.77 (0.20)	5050.8	10.77	4.46 (0.15)
	32	4805.5	99.86	2.71 (2.14)	4805.5	29.48	5.99 (0.13)	4805.5	12.13	6.10 (0.03)
6, 24h	16	5972.8	5889.23	4.47 (0.01)	5972.8	5463.50	20.12 (0.00)	5972.8	4271.71	68.19 (0.00)
	32	5854.9	5744.12	6.19 (0.01)	5854.9	5226.90	22.82 (0.02)	5854.9	3863.81	77.73 (0.00)
7, 24h	16	1140.8	74.63	1.68 (0.42)	1140.8	27.08	2.32 (0.09)	1140.8	13.56	2.59 (0.32)
	32	1156.6	81.85	3.62 (0.87)	1156.6	37.48	7.11 (0.18)	1156.6	33.49	8.01 (0.35)
8, 24h	16	12433.2	321.34	4.97 (0.20)	12433.2	213.57	6.77 (0.16)	12433.2	173.93	5.71 (0.44)
	32	11553.1	313.16	4.67 (0.45)	11553.1	218.26	4.88 (0.00)	11553.1	224.74	4.98 (0.13)
9, 24h	16	1168.5	22.79	1.00 (0.40)	1168.5	3.82	1.33 (0.03)	1168.5	0.83	1.40 (0.05)
	32	1057.9	23.84	1.94 (0.44)	1057.9	5.69	2.53 (0.11)	1057.9	3.79	2.59 (0.10)
10, 24h	16	7603.0	128.51	3.73 (0.57)	7603.0	61.45	4.60 (0.13)	7603.0	41.08	4.52 (0.41)
	32	7091.7	120.73	1.91 (0.02)	7091.7	65.64	2.32 (0.02)	7091.7	63.68	2.78 (0.41)
11, 24h	16	3754.2	92.43	2.12 (0.64)	3754.2	39.56	2.79 (1.08)	3754.2	33.95	2.79 (0.10)
	32	3489.3	217.79	1.97 (0.72)	3489.3	74.39	2.48 (0.19)	3489.3	61.79	2.40 (0.21)
12, 24h	16	1862.2	1462.40	6.59 (0.02)	1862.2	942.03	19.23 (0.00)	1862.2	428.08	39.68 (0.11)
	32	1770.7	1342.24	6.45 (0.00)	1770.7	830.80	18.42 (0.07)	1770.7	354.04	52.17 (0.20)
13, 24h	16	2036.9	36.11	1.08 (0.40)	2036.9	11.49	1.31 (0.07)	2036.9	8.32	1.42 (0.74)
	32	1826.9	37.23	1.06 (0.02)	1826.9	17.77	1.26 (0.18)	1826.9	15.75	1.26 (0.27)
14, 24h	16	1064.1	631.28	5.82 (0.00)	1064.1	337.30	14.60 (0.01)	1064.1	125.84	23.48 (0.10)
	32	1185.6	634.80	6.72 (0.01)	1185.6	310.04	15.28 (0.03)	1185.6	107.10	27.69 (0.49)
15, 24h	16	432.1	14.77	0.94 (0.84)	432.1	5.30	1.56 (0.14)	432.1	3.05	1.88 (0.30)
	32	421.3	15.41	1.30 (0.11)	421.3	6.78	1.51 (0.42)	421.3	5.49	1.61 (0.46)
16, 24h	16	6243.6	223.14	3.77 (0.20)	6243.6	160.44	4.53 (0.02)	6243.6	131.29	4.56 (0.11)
	32	6037.8	276.89	3.06 (0.02)	6037.8	253.44	3.39 (0.37)	6037.8	209.95	3.34 (0.10)
17, 24h	16	4513.1	48.04	1.13 (0.34)	4513.1	12.15	1.37 (0.73)	4513.1	7.01	1.42 (0.90)
	32	3857.9	51.19	1.75 (0.18)	3857.9	26.80	1.87 (0.32)	3857.9	21.06	1.92 (0.17)
18, 24h	16	4136.5	252.19	4.67 (0.05)	4136.5	51.14	10.35 (0.42)	4136.5	9.77	12.90 (0.43)
	32	4203.6	244.07	6.57 (0.70)	4203.6	60.18	6.22 (0.12)	4203.6	16.18	7.25 (0.36)
19, 24h	16	2051.3	25.86	0.99 (0.16)	2051.3	7.11	1.08 (0.19)	2051.3	4.51	1.09 (0.18)
	32	1872.7	25.74	1.48 (0.26)	1872.7	10.74	1.68 (0.07)	1872.7	10.48	1.73 (0.00)
20, 24h	16	3222.5	39.09	1.25 (0.15)	3222.5	8.31	2.05 (0.55)	3222.5	2.86	2.73 (0.13)
	32	3286.4	43.31	2.22 (1.59)	3286.4	11.06	4.39 (0.01)	3286.4	4.91	4.72 (0.07)
21, 24h	16	2526.6	32.42	1.30 (0.05)	2526.6	9.98	1.41 (0.03)	2526.6	8.24	1.41 (0.22)
	32	2806.9	36.07	1.60 (0.07)	2806.9	19.12	1.69 (0.07)	2806.9	15.32	1.70 (0.13)
22, 24h	16	2260.7	50.58	1.94 (0.28)	2260.7	17.68	2.93 (0.19)	2260.7	12.66	3.23 (0.41)
	32	2045.9	47.70	2.02 (0.59)	2045.9	15.10	2.32 (0.70)	2045.9	11.69	2.38 (0.82)
23, 24h	16	4397.1	18.09	1.25 (0.04)	4397.1	3.89	1.24 (0.12)	4397.1	2.10	1.24 (0.01)
	32	4600.0	23.30	1.57 (0.89)	4600.0	10.81	1.68 (0.53)	4600.0	8.34	1.37 (0.24)
24, 24h	16	4742.9	29.20	1.60 (0.06)	4742.9	7.58	1.72 (0.14)	4742.9	4.90	1.78 (0.05)
	32	4348.6	39.36	1.10 (0.37)	4348.6	18.99	1.14 (0.37)	4348.6	19.66	1.16 (0.23)

Table S3. (24–48h) Key diagnostics for the optimization-based velocity estimation algorithm for low ($n = 16$) and standard ($n = 32$) resolution meshes, and three values of the regularization parameter $\beta \in \{10^{-3}, 10^{-4}, 10^{-5}\}$ (set of columns) for the time intervals 24–48h. J_0: value of the objective functional without convective velocity i.e. $\phi = 0$, J_N: final value of the objective functional after algorithmic completion, $\max\phi_N$ (mm/h): maximal component of the optimal velocity field, $\Delta\phi\% = |\max\phi_{N-1} - \max\phi_N| / \max\phi_N 100\%$: relative difference between maximal component of the penultimate and ultimate velocity optimization iterate (in percent); low relative difference indicates that the numerical algorithm has converged.

ID	n	$\bar{\phi}$ (mm/h)	$\|\|c\|\|_0$	$\|\|c_1\|\|_0$	Δc (%)	$\bar{\phi}$ (mm/h)	$\|\|c\|\|_0$	$\|\|c_1\|\|_0$	Δc (%)	$\bar{\phi}$ (mm/h)	$\|\|c\|\|_0$	$\|\|c_1\|\|_0$	Δc (%)
3	16	0.27	50.01	45.40	10.16	0.51	46.14	45.40	1.63	0.60	45.06	45.40	0.76
	32	0.22	48.47	44.51	8.89	0.37	44.97	44.51	1.05	0.47	43.60	44.51	2.05
4	16	0.28	64.51	60.84	6.03	0.45	61.56	60.84	1.18	0.50	60.78	60.84	0.10
	32	0.24	64.68	61.36	5.41	0.32	61.76	61.36	0.65	0.33	61.37	61.36	0.03
7	16	0.20	53.26	50.82	4.80	0.34	51.31	50.82	0.95	0.40	50.95	50.82	0.26
	32	0.17	52.57	50.37	4.37	0.24	50.77	50.37	0.80	0.25	50.41	50.37	0.07
8	16	0.40	124.83	124.04	0.64	0.46	122.35	124.04	1.36	0.47	122.53	124.04	1.22
	32	0.28	125.82	125.25	0.45	0.31	123.62	125.25	1.30	0.31	123.68	125.25	1.25
9	16	0.14	71.20	70.04	1.67	0.18	70.14	70.04	0.16	0.19	69.98	70.04	0.08
	32	0.12	71.13	70.15	1.40	0.13	70.49	70.15	0.49	0.13	70.39	70.15	0.34
10	16	0.31	113.09	111.16	1.74	0.37	110.75	111.16	0.36	0.37	111.18	111.16	0.02
	32	0.22	114.14	112.87	1.12	0.25	112.69	112.87	0.16	0.25	112.39	112.87	0.42
11	16	0.23	112.90	111.50	1.26	0.29	111.40	111.50	0.09	0.29	111.39	111.50	0.10
	32	0.14	117.11	114.61	2.18	0.19	114.83	114.61	0.19	0.19	115.04	114.61	0.37
13	16	0.15	83.39	82.41	1.19	0.18	82.59	82.41	0.22	0.19	82.23	82.41	0.22
	32	0.12	84.11	83.11	1.20	0.13	83.66	83.11	0.66	0.13	83.57	83.11	0.55
15	16	0.09	47.94	47.26	1.43	0.11	47.51	47.26	0.52	0.13	47.36	47.26	0.21
	32	0.06	48.25	47.53	1.52	0.08	47.94	47.53	0.86	0.08	47.79	47.53	0.53
16	16	0.33	126.57	125.02	1.24	0.40	124.44	125.02	0.46	0.40	124.48	125.02	0.43
	32	0.24	126.05	125.54	0.41	0.24	125.93	125.54	0.31	0.24	125.88	125.54	0.27
17	16	0.19	106.83	105.63	1.14	0.22	105.85	105.63	0.22	0.23	105.66	105.63	0.03
	32	0.14	107.15	106.22	0.88	0.15	106.63	106.22	0.39	0.15	106.35	106.22	0.13
18	16	0.39	207.24	204.36	1.41	0.58	204.98	204.36	0.30	0.70	204.43	204.36	0.04
	32	0.35	213.71	211.22	1.18	0.50	211.76	211.22	0.26	0.55	211.51	211.22	0.14
19	16	0.13	98.99	98.14	0.86	0.16	98.32	98.14	0.18	0.16	98.23	98.14	0.09
	32	0.10	98.85	98.08	0.79	0.11	98.19	98.08	0.11	0.11	98.01	98.08	0.07
20	16	0.19	80.11	78.49	2.06	0.26	78.68	78.49	0.24	0.27	78.40	78.49	0.12
	32	0.16	79.89	78.35	1.97	0.19	78.52	78.35	0.22	0.20	78.52	78.35	0.21
21	16	0.14	112.86	112.70	0.14	0.16	112.44	112.70	0.24	0.16	112.42	112.70	0.25
	32	0.11	115.01	114.93	0.07	0.11	114.72	114.93	0.18	0.11	114.73	114.93	0.18
22	16	0.17	73.05	71.75	1.81	0.22	71.99	71.75	0.33	0.24	71.60	71.75	0.21
	32	0.14	72.63	71.41	1.71	0.17	71.89	71.41	0.67	0.17	71.59	71.41	0.26
23	16	0.11	221.48	221.29	0.08	0.12	221.29	221.29	0.00	0.12	221.25	221.29	0.02
	32	0.08	222.88	222.57	0.14	0.09	222.66	222.57	0.04	0.09	222.76	222.57	0.09
24	16	0.15	171.21	170.65	0.33	0.16	170.90	170.65	0.14	0.16	170.76	170.65	0.06
	32	0.10	176.80	176.37	0.24	0.11	176.53	176.37	0.09	0.11	176.42	176.37	0.03

Table S4. Evaluation of numerical accuracy of the estimated velocity fields and associated concentration fields for low ($n = 16$) and standard ($n = 32$) resolution meshes, and three values of the regularization parameter $\beta \in \{10^{-3}, 10^{-4}, 10^{-5}\}$ (set of columns) for the time intervals 24–48h. $\bar{\phi}$ (mm/h) is the brain-wide average velocity magnitude, $||c||_0$ the L^2-norm of the associated estimated concentration field, $||c_1||_0$ the L^2-norm of the corresponding target concentration field at end of the time interval (here ~48h), while Δc (in percent) gives the relative difference between the latter two values.

	ID	v (μm/min)	v_g	v_w	v_s
0	1	1.41	1.42	1.34	1.57
1	2	1.79	2.11	1.43	2.24
2	4	2.38	2.52	2.19	2.69
3	5	1.22	1.27	1.18	1.14
4	6	3.24	3.40	3.01	4.33
5	7	3.24	3.45	2.86	5.06
6	8	2.81	3.08	2.47	2.77
7	9	3.27	3.44	3.02	3.41
8	10	1.78	1.85	1.69	1.78
9	11	2.16	2.34	1.90	3.04
10	12	2.91	3.12	2.61	3.54
11	13	3.08	3.33	2.68	4.92
12	14	2.63	2.88	2.33	4.01
13	15	4.06	4.29	3.71	5.94
14	16	1.89	1.88	1.89	2.14
15	17	1.62	1.79	1.43	1.69
16	18	1.37	1.27	1.48	1.54
17	19	2.05	2.14	1.91	2.75
18	20	2.45	2.57	2.26	3.84
19	21	1.89	2.09	1.61	2.83
20	22	2.18	2.29	2.04	2.53
21	24	1.60	1.73	1.43	1.52
Ref. (n=16)		2.40 ± 0.82	2.54 ± 0.89	2.10 ± 0.72	2.98 ± 1.34
Dep. (n=6)		2.11 ± 0.57	2.28 ± 0.60	1.89 ± 0.50	2.92 ± 1.29
Total (n=22)		2.32 ± 0.75	2.48 ± 0.81	2.11 ± 0.67	2.97 ± 1.30

(a) 6–24h

	ID	v (μm/min)	v_g	v_w	v_s
0	3	6.14	7.10	5.07	4.42
1	4	5.29	5.93	4.54	4.61
2	7	3.97	4.35	3.42	3.97
3	8	5.19	6.06	4.18	3.32
4	9	2.21	2.52	1.79	1.57
5	10	4.15	4.55	3.74	1.99
6	11	3.21	3.47	2.90	2.51
7	13	2.12	2.20	2.05	1.09
8	15	1.25	1.37	1.13	0.86
9	16	3.97	4.30	3.62	3.11
10	17	2.48	2.64	2.32	1.49
11	18	8.39	8.14	8.59	11.42
12	19	1.81	1.84	1.78	1.58
13	20	3.23	3.58	2.81	4.13
14	21	1.87	2.03	1.68	2.06
15	22	2.76	3.01	2.48	2.06
16	23	1.46	1.47	1.43	1.68
17	24	1.79	1.90	1.68	1.30
Ref. (n=11)		4.23 ± 1.98	4.62 ± 2.03	3.77 ± 1.98	3.62 ± 2.85
Dep. (n=7)		2.11 ± 0.59	2.24 ± 0.69	1.96 ± 0.47	1.90 ± 1.03
Total (n=18)		3.41 ± 1.89	3.69 ± 2.01	3.07 ± 1.79	2.95 ± 2.43

(b) 24–48h

Table S5. Subject-specific average velocities v denotes the brain-wide average flow speed (velocity magnitude, in μm/min), while v_g, v_w and v_s denote the cerebral cortex average, subcortical white matter average and brain stem average flow speed, respectively. Note the use of μm/min here and in Results versus mm/h for Table SM3. See text in Section S3.2 for further details.

Table S6. Properties of the velocity field ϕ obtained with inverse modelling for two different preprocessing methods (using DTI & T_1 map vs using study averaged constants for D and T_1), averaged over $n = 15$ subjects where both DTI and T_1 map is available.

	time interval	6-24 h		24-48 h	
	method	T1 & DTI	avg. T1 & avgD	T1 & DTI	avg. T1 & avgD
Flow speed $\overline{\phi}$ (μm/min)	brain	2.43	2.50	3.18	3.26
	Cerebral cortex	2.58	2.66	3.38	3.48
	subcortical white matter	2.19	2.27	2.92	2.99
	brainstem	3.53	3.34	3.20	3.03
Fluid influx rate $\frac{1}{\lvert\Omega\rvert}\int_\Omega \nabla \cdot \phi \, \mathrm{d}x$ ($10^{-4}\mathrm{min}^{-1}$)	Brain-wide	0.95	0.95	2.67	2.72
	Cerebral cortex	2.55	2.58	2.92	2.93
	Subcortical white matter	-1.14	-1.13	2.32	2.44
	brain stem	3.10	2.37	3.54	3.00

Table S7. Dependence of optimal dispersion-reaction parameters on data integration choices. Time step = 10 min.

method	avg. T_1 & avg. D	filtered T_1 & DTI	avg. T_1 & avg. D	filtered T_1 & DTI
subject ID	α		r (10^{-4} min^{-1})	
4	3.372	3.423	22.856	23.691
7	2.996	3.070	25.835	35.239
9	3.858	3.451	34.087	34.434
10	2.142	1.855	23.660	23.623
11	4.546	4.956	57.514	60.000
12	5.234	4.784	58.365	60.000
13	4.076	3.555	40.029	41.421
14	4.565	4.223	35.228	40.018
15	4.364	4.044	54.684	59.334
17	2.514	2.512	30.045	29.701
18	1.497	1.299	15.533	17.026
20	3.736	3.876	10.817	11.009
21	3.000	4.722	12.491	26.952
22	2.500	2.472	26.911	28.840
24	1.120	1.305	13.942	22.025
mean ± std	3.301 ± 1.152	3.303 ± 1.162	30.800 ± 15.418	34.221 ± 14.955

Table S8. Dependence of optimal parameters on time steps size. In most subjects, the optimal parameters change less than 10% between time steps 20 and 10 min, and we hence did not run computationally expensive simulations with a time step of 5 minutes in these subjects.

pat	dt (min)	α	r (10^{-4} min^{-1})	J_p	J_0	J_F
1	30	3.8	40	0.29	0.29	0.25
	20	3.9	41	0.29	0.29	0.25
	10	3.9	40	0.29	0.29	0.25
	5					
2	30	2.8	50	0.37	0.39	0.35
	20	2.8	51	0.37	0.39	0.34
	10	2.8	49	0.37	0.39	0.34
	5					
3	30	1.2	8	0.81	0.83	0.80
	20	3.0	14	0.50	0.46	0.46
	10	10.0	44	0.43	0.38	0.35
	5	10.0	47	0.43	0.35	0.35
4	30	2.1	14	0.46	0.44	0.42
	20	3.0	20	0.31	0.27	0.25
	10	3.4	23	0.26	0.21	0.19
	5	3.4	23	0.26	0.21	0.20
5	30	1.5	23	0.24	0.32	0.20
	20	1.4	21	0.24	0.32	0.20
	10	1.5	22	0.24	0.32	0.20
	5					
6	30	4.2	7	0.53	0.46	0.46
	20	5.9	10	0.37	0.29	0.27
	10	7.1	12	0.35	0.26	0.23
	5	7.0	11	0.34	0.26	0.23
7	30	1.8	15	0.40	0.40	0.37
	20	2.7	23	0.30	0.27	0.25
	10	3.0	26	0.28	0.26	0.24
	5	3.0	26	0.28	0.26	0.24
8	30	3.6	19	0.46	0.40	0.39
	20	5.5	31	0.41	0.34	0.32
	10	6.4	37	0.40	0.33	0.31
	5	6.6	39	0.40	0.33	0.31
9	30	1.9	16	0.67	0.66	0.63
	20	3.4	28	0.41	0.38	0.36
	10	3.9	34	0.36	0.32	0.30
	5	3.8	33	0.36	0.32	0.30
10	30	2.2	24	0.28	0.25	0.16
	20	2.3	25	0.28	0.25	0.16
	10	2.1	24	0.28	0.25	0.16
	5					
11	30	2.4	30	0.52	0.51	0.45
	20	4.0	50	0.42	0.39	0.33
	10	4.5	58	0.40	0.37	0.31
	5	4.8	62	0.40	0.37	0.31
12	30	3.2	33	0.42	0.38	0.35
	20	4.3	46	0.38	0.33	0.30
	10	5.2	58	0.38	0.33	0.30
	5	5.2	59	0.37	0.33	0.30
13	30	3.6	36	0.25	0.21	0.17
	20	4.1	40	0.26	0.21	0.17
	10	4.1	40	0.26	0.21	0.17
	5					

(a)

pat	dt (min)	α	r (10^{-4} min^{-1})	J_p	J_0	J_F
14	30	4.5	36	0.39	0.34	0.32
	20	4.7	36	0.39	0.33	0.31
	10	4.6	35	0.39	0.33	0.31
	5					
15	30	4.1	51	0.31	0.28	0.24
	20	4.5	57	0.32	0.28	0.24
	10	4.4	55	0.31	0.28	0.24
	5					
16	30	2.7	24	0.28	0.24	0.20
	20	2.7	23	0.27	0.24	0.20
	10	2.9	25	0.28	0.24	0.20
	5					
17	30	2.3	27	0.26	0.25	0.17
	20	2.5	30	0.26	0.25	0.17
	10	2.5	30	0.26	0.25	0.17
	5					
18	30	1.5	16	0.24	0.25	0.15
	20	1.5	16	0.24	0.26	0.15
	10	1.5	16	0.24	0.26	0.15
	5					
19	30	2.9	30	0.34	0.30	0.24
	20	3.2	33	0.34	0.30	0.24
	10	3.3	33	0.34	0.30	0.24
	5					
20	30	3.0	9	0.28	0.23	0.23
	20	3.0	9	0.28	0.23	0.23
	10	3.7	11	0.28	0.23	0.23
	5	3.7	11	0.28	0.23	0.23
21	30	3.0	12	0.36	0.31	0.31
	20	3.0	12	0.36	0.32	0.31
	10	3.0	12	0.36	0.31	0.31
	5					
22	30	2.9	33	0.29	0.28	0.23
	20	2.6	28	0.29	0.28	0.23
	10	2.5	27	0.29	0.28	0.23
	5					
23	30	1.1	14	0.31	0.34	0.18
	20	1.1	14	0.31	0.33	0.17
	10	1.1	14	0.31	0.33	0.18
	5					
24	30	2.7	25	0.28	0.27	0.22
	20	3.0	29	0.28	0.28	0.22
	10	2.5	24	0.28	0.28	0.22
	5	2.6	24	0.28	0.27	0.22

(b)

Table S9. Optimal enhanced diffusion-local clearance parameters.

Subject ID	sleep group?	best α	best r (10^{-4}min^{-1})	Rel. L^2-error with ($\alpha = 1, r = 0$)	Rel. L^2-error with best (α, r)	red. in rel L^2-error (%)	time steps required for convergence
1	True	3.9	40	0.29	0.25	13	288
2	True	2.8	49	0.37	0.34	7	288
4	True	3.4	23	0.26	0.20	24	576
5	True	1.5	22	0.24	0.20	15	288
6	True	7.0	11	0.34	0.23	32	576
7	True	3.1	26	0.28	0.24	16	576
8	True	6.6	39	0.40	0.31	23	576
9	True	3.8	34	0.36	0.30	16	576
10	True	2.1	24	0.28	0.16	42	288
11	True	4.8	62	0.40	0.31	22	576
12	True	5.2	59	0.37	0.30	21	576
13	False	4.1	40	0.26	0.17	35	288
14	True	4.6	35	0.39	0.31	19	288
15	True	4.4	55	0.31	0.24	22	288
16	True	2.9	25	0.28	0.20	27	288
17	False	2.5	30	0.26	0.17	35	288
18	True	1.5	16	0.24	0.15	37	288
19	False	3.3	33	0.34	0.24	29	288
20	False	3.7	11	0.28	0.23	19	576
21	False	3.0	12	0.36	0.31	15	288
22	True	2.5	27	0.29	0.23	20	288
23	False	1.1	14	0.31	0.18	43	288
24	False	2.6	24	0.28	0.22	22	576
